# Identification of basic/helix-loop-helix transcription factors reveals candidate genes involved in anthocyanin biosynthesis from the strawberry white-flesh mutant

**DOI:** 10.1038/s41598-018-21136-z

**Published:** 2018-02-09

**Authors:** Fengli Zhao, Gang Li, Panpan Hu, Xia Zhao, Liangjie Li, Wei Wei, Jiayue Feng, Houcheng Zhou

**Affiliations:** 1 Zhengzhou Fruit Research Institute, Chinese Academy of Agricultural Sciences, Zhengzhou, China; 2 Key Laboratory of Fruit Breeding Technology, Ministry of Agriculture of China, Zhengzhou, China; 3 College of Horticulture, Northwest A&F University, Yangling, China

## Abstract

As the second largest transcription factor family in plant, the basic helix-loop-helix (bHLH) transcription factor family, characterized by the conserved bHLH domain, plays a central regulatory role in many biological process. However, the bHLH transcription factor family of strawberry has not been systematically identified, especially for the anthocyanin biosynthesis. Here, we identified a total of 113 bHLH transcription factors and described their chromosomal distribution and bioinformatics for the diploid woodland strawberry *Fragaria vesca*. In addition, transcription profiles of 113 orthologous bHLH genes from various tissues were analyzed for the cultivar ‘Benihoppe’, its white-flesh mutant ‘Xiaobai’, and the ‘Snow Princess’ from their fruit development to the ripening, as well as those under either the ABA or Eth treatment. Both the RT-PCR and qRT-PCR results show that seven selected *FabHLH* genes (*FabHLH17*, *FabHLH25*, *FabHLH27*, *FabHLH29*, *FabHLH40*, *FabHLH80*, *FabHLH98*) are responsive to the fruit anthocyanin biosynthesis and hormone signaling according to transcript profiles where three color modes are observed for strawberry’s fruit skin and flesh. Further, prediction for the protein interaction network reveals that four bHLHs (*FabHLH25*, *FabHLH29*, *FabHLH80*, *FabHLH98*) are involved in the fruit anthocyanin biosynthesis and hormone signaling transduction. These bioinformatics and expression profiles provide a good basis for a further investigation of strawberry bHLH genes.

## Introduction

The basic helix-loop-helix (bHLH) proteins, named by their signature conserved domain, form a large superfamily of transcription factor. They are widely distributed from yeast to human^1,2^ and play a central role in many different functions in the development of animals and plants^3,4^. Typically, a bHLH domain consists of ~60 amino acids with two functionally distinct regions, and it comprises a stretch of about 13–17 hydrophilic basic amino acids at the N-terminal (basic region), followed by two regions of hydrophobic residue α-helix separated by an intervening loop (HLH region)^5^. The basic region, which contains six typical residues with a highly conserved HER motif (His-Glu-Arg) and is thus relevant to its binding to DNA sequences, allows HLH proteins to specifically adhere to the E-box (5′-CANNTG-3′) or the variant G-box (5′-CACGTG-3′), where N corresponds to any nucleotide^3,6–8^. The HLH region functions as a dimerization domain which promotes the formation of homodimers or heterodimers complex, and has been found to be highly conserved in organisms^3,5,9,10^. Furthermore, the bHLH motifs have been characterized to be able to modulate gene expression by binding to DNA sequences and further participate in plant development^3,11^.

With the completion of the plant genome sequencing, a large number of bHLH sequences are identified. Recently, more and more bHLH gene families have been located with the investigation of their functions for plants, including *Arabidopsis* (*Arabidopsis thaliana*)^9,12^, tomato (*Solanum lycopersicum*)^3,4^, common bean (*Phaseolus vulgaris*)^7^, apple (*Malus* × *domestica*)^8^, Chinese cabbage (*B*. *rapa* ssp. *pekinensis*)^13^, cotton (*Gossypium*)^14^. According to their bioinformatics and evolutionary relationships^2–4,7,8,13^, bHLH genes are classified into 15–26. For *Arabidopsis*, 162 bHLH genes have been identified from genome sequences and consequently been divided into 26 subfamilies according to the topology of trees, clade support values, branch lengths, and visual inspection^2^. In addition, for the ‘Golden Delicious’ apple, 188 *MdbHLH* (*Malus* × *domestica* bHLH) transcription factors are sorted out and classified into 18 subfamilies^8^. Furthermore, transcription factors belonging to the same subfamily show similar structure, motif and protein function in plant^1,9^.

bHLH transcription factors are important regulators in plant physiology, such as anthocyanin biosynthesis^2,10,15^, biotic and abiotic stress^12,16,17^, organ development^1,12,18^, etc. To date, certain types of plant *bHLH* genes have been studied in-depth, providing insights into their biochemical functions^5^ and central roles of transcription factors. For example, genes, located in the IIIf subfamily of bHLH for *Arabidopsis*, have been proved to be involved in both the flavonoid biosynthesis and trichome formation^5^. Molecular interaction of TT8 (Transparent testa 8), GL3 (Glabra 3) and EGL3 (Enhancer of Glabra 3) from the IIIf subfamily with TTG1 (Transparent testa glabra1, WD40 protein family) and MYB (myeloblastosis protein, MYB protein family) forms a MBW (MYB-bHLH-WD40) complex, which regulates genes in the anthocyanin biosynthesis for *Arabidopsis* and tomato^6,19–22^. Besides, genes from bHLH III(d + e) subfamily have been demonstrated to be able to regulate the JA signal pathway to enhance the plant defense and promote the anthocyanin biosynthesis^23–27^. The underlying mechanism for the former is that MYC2 (myelocytomatosis 2) is crucial to the plant growth and thus may enhance disease resistance for apple^28^. For the latter, low temperature facilitates the expression of *MdbHLH3*, which regulates anthocyanin accumulation and fruit coloration for apple^27,29^.

Strawberry (*Fragaria × ananassa* Duch.) is well recognized universally as a delicious and healthy food^30^. In recent years, white strawberry is more and more favored by consumers, such as ‘Xiaobai’^31^, ‘Snow Princess’ and ‘Tokun’ varieties. As a result, numerous researchers have been casting their eyes on the fruit ripening, ABA (abscisic acid) signaling pathway^32–36^ and anthocyanin biosynthesis^37,38^. Roles of MYB transcription factors have been highlighted in the anthocyanin biosynthesis^20,37,38^, while very few reports on bHLH transcription factors have been made^38–40^ and they are mostly limited to the single bHLH. For example, anthocyanin biosynthesis is essentially regulated by the *FvDFR* (*F. vesca* DFR, dihydroflavonol 4-reductase) and *FvUFGT* (*F. vesca* UFGT, 3-O-glucosyltransferase), which can be activated by *FvbHLH33* (*F. vesca* bHLH33) with the co-expression of *FvMYB10* (*F. vesca* MYB10)^39^. Moreover, FabHLH3 (*F. ananassa* bHLH3) and FabHLH3∆ (encode putative negative regulator), by interacting with the four MYBs, are found to be involved in the proanthocyanidins biosynthesis for strawberry^38^. In order to systematically explore the molecular basis of bHLH from all of FvbHLHs involved in the anthocyanin biosynthesis and hormone response pathway, we will first analyze the bioinformation of 113 bHLH genes for the diploid woodland strawberry, *F. vesca*, and reveal their structure, evolution and function. Furthermore, we will study the transcript profiles of *FabHLH* genes from various tissues for the cultivar ‘Benihoppe’, its white flesh mutant ‘Xiaobai’, and the ‘Snow Princess’ from their fruit development to the ripening period, as well as those under either the ABA or Eth (ethephon) treatment. We finally discover that seven *FabHLHs* are crucial to the anthocyanin biosynthesis and fruit ripening for the strawberry fruit. We hope that this work will serve as a solid foundation for further investigations into functions of bHLH genes for the anthocyanin biosynthesis.

## Results

### Identification and annotation of bHLH transcription factors in strawberry

**Figure 1:**
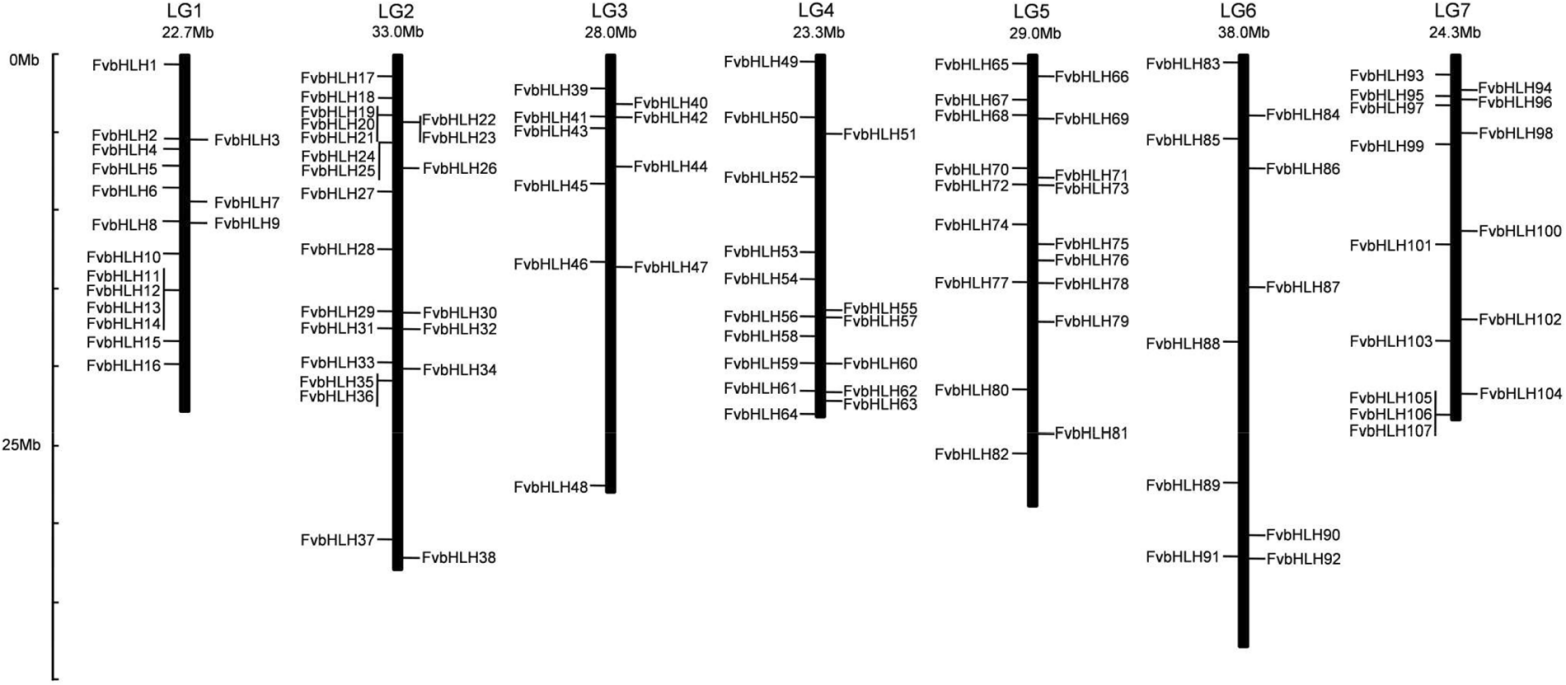
Chromosomal distributions of *FvbHLH* genes. The name on the side of each chromosome corresponds to the approximate location of each bHLH gene.

To identify bHLH transcription factors for *F. vesca*, a total of 166 bHLH members for strawberry via the BLAST-P (Basic Local Alignment Search Tool) search in the database of NCBI (National Center for Biotechnology Information) were obtained by comparing with the 112 strawberry bHLH amino acid sequences from the Plant Transcription Factor Database for the diploid woodland strawberry accession Hawaii-4 genome. Subsequently, to verify the reliability of the selection, a survey was conducted to confirm the presence of the conserved bHLH domain in protein sequences using the online CDD (Conserved Domains Database), SMART (Simple Modular Architecture research tool), and InterProScan database. The unique hits are kept, and duplications and similar DNA or protein sequences (with several bases different) are ruled out with only one of them left^10^. For example, there are four alternative variants for the sequence of *FvbHLH64*, only the longest variant is kept for the further analysis. In the end, 113 out of the 166 FvbHLH members are eventually selected (Table 1) out, forming the bHLH family for strawberry. The first 107 genes are renamed from *FvbHLH1 to FvbHLH107* according to their distributions on the chromosome 1–7 from NCBI database^8,41^ (Table 1; Fig. 1). In particular, the left 6 on unknown chromosome are renamed from *FvbHLH108* to *FvbHLH113* by their position value from the minimum to the maximum(Table 1). The acquired 113 bHLH genes will be further used to study their bioinformation and biofunction, specially for the anthocyanin biosynthesis.

**Table 1:** Details of bHLH gene family for strawberry.

Name	Accession no.	length(bp)	No. of aa	Mw(Da)	pI	Chr.	Location	Group
FvbHLH1	XM_004287073	1999	459	39465.8	6.67	LG1	774071..777509	VII(a + b)
FvbHLH2	XM_011472368	1508	353	39258.2	4.83	LG1	5628786..5630945	III(a + c)
FvbHLH3	XM_011472373	999	328	35846.2	5.09	LG1	5633593..5635348	III(a + c)
FvbHLH4	XM_004288907	2261	609	68051	7.62	LG1	6270695..6273919	IVd
FvbHLH5	XM_011462840	1688	422	46054.2	4.71	LG1	7257743..7260881	XI
FvbHLH6	XM_004287765	1722	321	35480.6	7.04	LG1	8558473..8560804	IVb
FvbHLH7	XM_011464708	1979	378	41465.2	9.25	LG1	9553758..9555736	VIII
FvbHLH8	XM_004287975	1584	376	40793.2	5.27	LG1	10770968..10773625	VII(a + b)
FvbHLH9	XM_004287983	1126	250	27603.6	9.81	LG1	10839398..10840523	VIIIb
FvbHLH10	XM_004288109	1417	335	37562.9	6.2	LG1	12901813..12903720	Ia
FvbHLH11	XM_011468187	1629	351	39316.2	6.03	LG1	15128707..15131319	IVa
FvbHLH12	XM_004288253	1878	352	39142.8	6.13	LG1	15140049..15142329	IVa
FvbHLH13	XM_004288264	1519	365	40767.6	4.64	LG1	15309610..15311394	IIIb
FvbHLH14	XM_004289363	1400	296	33300.1	6.19	LG1	15852165..15853564	VIIIb
FvbHLH15	XM_004288393	968	240	27430.5	9.35	LG1	18393887..18395702	Ib
FvbHLH16	XM_004289479	880	216	23826	10.2	LG1	19941466..19942513	Vb
FvbHLH17	XM_004289620	1724	422	46479.1	6.06	LG2	1449671..1452519	XII
FvbHLH18	XM_004289703	1445	273	29597.9	7.62	LG2	2948918..2950935	X
FvbHLH19	XM_004289751	2926	454	48833.1	8.64	LG2	3904357..3908604	VII(a + b)
FvbHLH20	XM_004292083	810	269	30282.7	4.95	LG2	3955128..3956367	VIIIc
FvbHLH21	XM_011461385	1084	349	38855.1	5.38	LG2	3963999..3965470	VIIIc
FvbHLH22	XM_011459605	1565	332	37043.7	8.94	LG2	4390466..4392690	IVa
FvbHLH23	XM_011459618	1462	325	35939.1	5.95	LG2	4612200..4615547	IVb
FvbHLH24	XM_004289847	1561	292	31061.1	5.91	LG2	5528604..5532429	XI
FvbHLH25	XM_011459681	2241	504	55378.5	5.77	LG2	5784856..5787096	III(d + e)
FvbHLH26	XM_011459763	2595	518	56543.3	5.83	LG2	7260709..7265049	VII(a + b)
FvbHLH27	XM_004292295	2144	544	59176.8	6.42	LG2	8870693..8873438	XII
FvbHLH28	XM_004290363	1260	244	27544	6.2	LG2	12507957..12509504	Ib
FvbHLH29	XM_004290615	2955	702	77736.9	5.54	LG2	16556642..16562043	IIIf
FvbHLH30	XM_004290623	1197	296	33052.3	5.3	LG2	16628469..16632121	VIII
FvbHLH31	XM_011460379	2134	583	66027.7	5.22	LG2	17537839..17540937	IIIb
FvbHLH32	XM_011460381	2473	674	76146.1	5.16	LG2	17543996..17547612	IIIb
FvbHLH33	XM_011461514	1208	381	42148.2	5.05	LG2	19839411..19840894	VIIIc
FvbHLH34	XM_004290949	2921	430	46646.8	5.58	LG2	20151639..20155328	XII
FvbHLH35	XM_011460622	1425	324	35244.1	5.88	LG2	20942790..20945565	VII(a + b)
FvbHLH36	XM_004292764	1164	244	27373	7.96	LG2	21026021..21027184	VIIIb
FvbHLH37	XM_011461186	1047	262	29141.9	6.61	LG2	30979646..30981384	Vb
FvbHLH38	XM_004291800	2076	468	50979.6	7.79	LG2	32278737..32282900	X
FvbHLH39	XM_004293556	1718	244	27140	4.9	LG3	2254773..2258017	XIV
FvbHLH40	XM_011461973	1389	93	10385.6	5.07	LG3	3263115..3264716	XV
FvbHLH41	XM_011462057	1986	484	51788	5.46	LG3	4011392..4014350	X
FvbHLH42	XM_011462059	1003	216	24434	5.44	LG3	4026675..4028036	III(a + c)
FvbHLH43	XM_011462115	1885	286	32432.7	5.44	LG3	4881126..4883099	VIII
FvbHLH44	XM_011462301	2375	262	28591.3	7.74	LG3	7317885..7322470	Vb
FvbHLH45	XM_004293965	1075	187	20972.7	7.02	LG3	8396475..8397892	Ib
FvbHLH46	XM_004294266	2082	448	49286.4	6.4	LG3	13388482..13391583	X
FvbHLH47	XM_004294288	1229	231	25421	8.44	LG3	13637964..13641643	IVc
FvbHLH48	XM_004295162	3075	431	47638.7	6.09	LG3	27564756..27568579	IX
FvbHLH49	XM_004296310	1113	231	25855.3	6.02	LG4	53766..56495	IVc
FvbHLH50	XM_004296450	1106	244	27632.5	5.46	LG4	4132264..4135925	III(a + c)
FvbHLH51	XM_004296502	1059	94	10600	7.93	LG4	5165859..5167450	XV
FvbHLH52	XM_004296662	1799	350	37266.4	8.78	LG4	7927705..7935898	IX
FvbHLH53	XM_004296894	1024	202	22735.5	9.27	LG4	12705986..12707777	Ia
FvbHLH54	XM_004296937	2317	550	59476.9	6.3	LG4	14464166..14467207	XII
FvbHLH55	XM_004297098	1619	345	38323.1	6.07	LG4	16335664..16338026	Vb
FvbHLH56	XM_004297136	3010	550	59018.9	5.25	LG4	16797062..16800740	XII
FvbHLH57	XM_004297147	1599	331	36302.8	5.95	LG4	16909459..16912235	Va
FvbHLH58	XM_004297270	1047	98	11187.5	9.03	LG4	18127224..18128727	XV
FvbHLH59	XM_004297447	2201	535	58210	5.81	LG4	19786891..19789971	IIIb
FvbHLH60	XM_004298488	1789	339	37867.3	7.12	LG4	19872469..19875066	XII
FvbHLH61	XM_011465048	1910	436	48434.6	6.18	LG4	21544358..21546437	VIIIb
FvbHLH62	XM_004298520	1812	464	52734.2	6.36	LG4	21671222..21673829	III(a + c)
FvbHLH63	XM_004297644	1599	275	29997.3	6.02	LG4	22264260..22267220	XII
FvbHLH64	XM_011465190	2108	533	58065.7	6.78	LG4	23023913..23027032	orphans
FvbHLH65	XM_004300691	1309	262	29626.9	6.42	LG5	686954..688474	Ib
FvbHLH66	XM_004298754	3254	636	71225.1	5.51	LG5	1485513..1490045	IIIf
FvbHLH67	XM_004298887	836	191	21670.1	9.72	LG5	2965347..2966500	Ib
FvbHLH68	XM_004300913	1850	412	45859.4	6.39	LG5	4025201..4029399	orphans
FvbHLH69	XM_004299007	1180	276	31128.1	6.45	LG5	4234865..4236694	XII
FvbHLH70	XM_004299222	2515	571	62714.7	8	LG5	7347973..7353265	Va
FvbHLH71	XM_004301080	1492	394	44179.6	7.83	LG5	7937035..7939413	VII(a + b)
FvbHLH72	XM_004299332	1209	246	26920.3	5.99	LG5	8368043..8370357	IVb
FvbHLH73	XM_004299336	984	92	10426.7	9.17	LG5	8419238..8420695	XV
FvbHLH74	XM_004299574	1299	274	29662.9	5.68	LG5	10962434..10965206	XII
FvbHLH75	XM_004299639	1500	340	38514.2	6.16	LG5	12157286..12159348	Ia
FvbHLH76	XM_004301340	1020	339	38024.9	4.71	LG5	13269137..13271499	VIIIc
FvbHLH77	XM_011466454	1192	224	24295.2	5.29	LG5	14535826..14540081	IX
FvbHLH78	XM_004299778	1442	354	40009.1	4.85	LG5	14623387..14625157	IIIb
FvbHLH79	XM_011466590	1909	386	41948.8	6.7	LG5	17225517..17229489	X
FvbHLH80	XM_004300191	2690	682	74856.2	5.52	LG5	21462279..21464968	III(d + e)
FvbHLH81	XM_004300360	1268	321	35418.5	6.67	LG5	24315240..24317523	Ia
FvbHLH82	XM_004300469	1196	295	32841.5	5.45	LG5	25509922..25511575	XII
FvbHLH83	XM_004302005	2095	339	36374.6	6.07	LG6	538981..543382	XI
FvbHLH84	XM_004302277	2778	710	76643.7	6.08	LG6	3989352..3994265	VII(a + b)
FvbHLH85	XM_004302404	1427	342	39041.4	5.67	LG6	5477305..5478975	Ia
FvbHLH86	XM_011470140	525	174	19761.3	8.71	LG6	7363269..7363793	VIIIb
FvbHLH87	XM_011468604	997	230	26241.7	6.14	LG6	14930398..14931670	Ib
FvbHLH88	XM_004305478	1408	298	32732.1	6.59	LG6	18431373..18433464	VIIIc
FvbHLH89	XM_004303853	2182	540	59336.8	7.66	LG6	27360228..27363650	VII(a + b)
FvbHLH90	XM_004304146	1469	322	36058.5	5.7	LG6	30729849..30733592	Va
FvbHLH91	XM_004304266	1728	319	35277.2	8.32	LG6	32166984..32169818	IX
FvbHLH92	XM_004304269	1670	352	38982.9	5.97	LG6	32238590..32243935	XII
FvbHLH93	XM_004308165	1937	429	47554.5	8.19	LG7	1392304..1395438	IX
FvbHLH94	XM_011470561	1430	294	33574.5	6.15	LG7	2332756..2334886	VIII
FvbHLH95	XM_004306563	2160	420	46844.5	6.1	LG7	2743026..2745885	Ia
FvbHLH96	XM_004306579	1955	491	54529.8	5.03	LG7	2955020..2956974	III(d + e)
FvbHLH97	XM_004306609	3013	616	67483	6.11	LG7	3395170..3398461	III(d + e)
FvbHLH98	XM_004308329	2615	643	71423.8	5.42	LG7	5102380..5106265	IIIf
FvbHLH99	XM_011470775	1286	361	39894.6	6.6	LG7	5799342..5800960	Ia
FvbHLH100	XM_004308575	2010	465	52012.7	5.61	LG7	11372618..11375286	II
FvbHLH101	XM_011471173	1907	243	27413.7	5.92	LG7	12278020..12280123	Ib
FvbHLH102	XM_004307508	1708	446	50855	6.11	LG7	17017617..17022889	X
FvbHLH103	XM_004307626	1299	261	28984	9.04	LG7	18389468..18391089	Vb
FvbHLH104	XM_004309112	1767	366	40022.1	5.39	LG7	21789731..21792322	Ia
FvbHLH105	XM_004308085	1469	254	28708.4	5.51	LG7	23053917..23055551	Ib
FvbHLH106	XM_011472396	792	196	21478.6	9.61	LG7	23057596..23058575	Ib
FvbHLH107	XM_004308087	1609	261	29145.2	9.42	LG7	23060616..23062531	Ib
FvbHLH108	XM_004309700	2236	365	40673.8	7.06	Un	17881..23082	Ib
FvbHLH109	XM_004309608	1230	245	26615.1	5.83	Un	32335..34765	IVc
FvbHLH110	XM_004309546	1181	166	18402	9.1	Un	56243..60570	X
FvbHLH111	XM_011459272	3679	780	85359.2	5.24	Un	147469..152520	XIII
FvbHLH112	XM_011459212	949	175	19447.2	5.77	Un	1751808..1753619	XII
FvbHLH113	XM_004310203	1028	171	19329.6	11.5	Un	1966158..1967185	XV

Accession numbers are available in the National center for Biotechnology Information database. Mw, molecular weight; pI, isoelectric point.

### Phylogenetic analysis and multiple sequence alignments of the strawberry FvbHLH proteins

**Figure 2:**
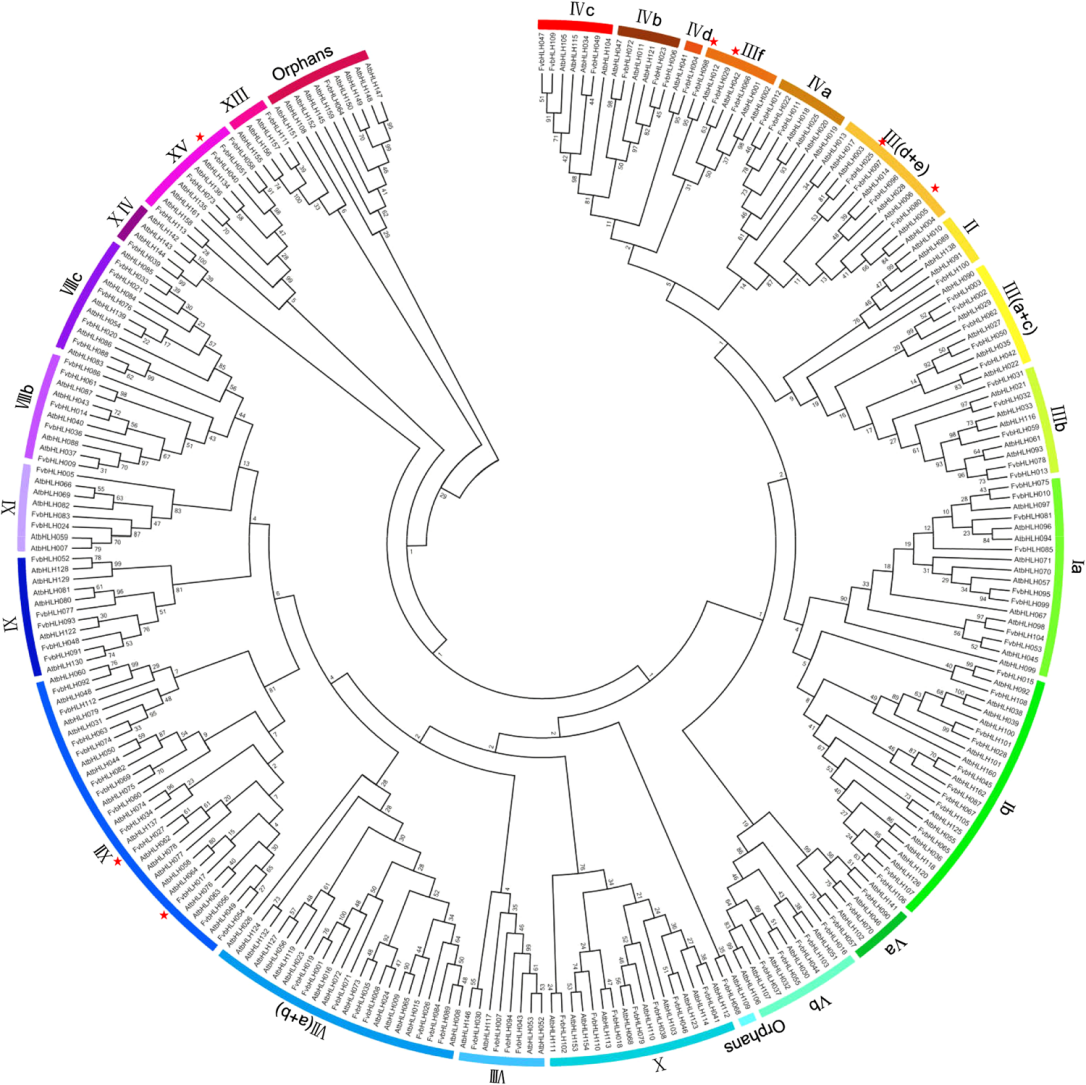
Phylogenetic tree constructed from the neighbor-joining method using the bHLH transcription factor domain for strawberry. Genes marked by the red asterisk indicates the seven candidates of FvbHLH involved in the anthocyanin biosynthesis.

Reflecting on the past researches, the exact number of the classified subfamily for bHLH proteins has barely been reported^8^. To investigate the classification and evolution as well as to gain insights into the potential function of FvbHLH proteins for strawberry, we constructed a phylogenetic tree (Fig. 2) for the 113 *FvbHLHs* from *F. vesca* and 158 *AtbHLHs* from *Arabidopsis*. 26 of bHLH subfamilies are further classified according to the nomenclature protocol proposed by Heim *et al*.^5^, with some modifications. For example, I(a + b) is divided into Ia and Ib, and IIIa and IIIc are combined into III(a + c); bHLHs that are not located in any of the 24 subfamilies are classified as “orphans” (Fig. 2). We find that FvbHLH protein is persistently present in all subfamilies and the number of it varies hugely from subfamily to subfamily. For instance, each of the smallest group II, IVd, XIII and XIV contains one *FvbHLH* gene, while the largest clade group XII contains twelve. Consequently, the classification of *bHLH* genes provides an evidence for relationships among genes during their evolution.

**Figure 3:**
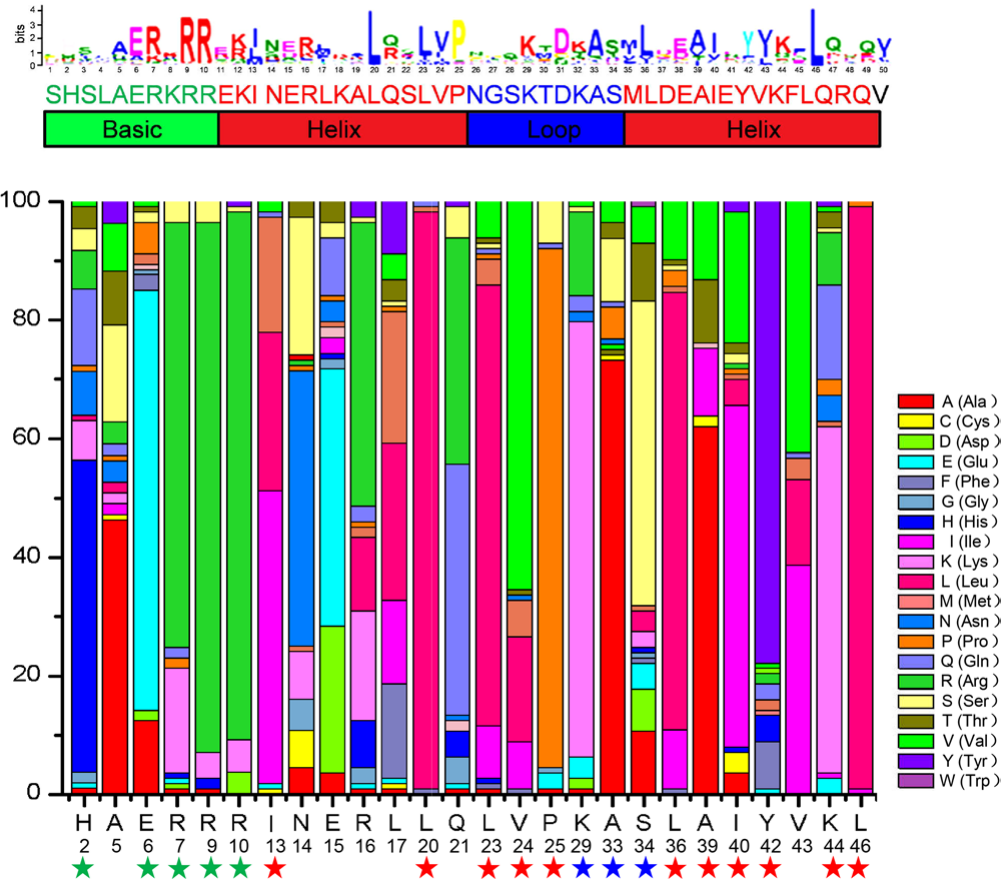
The characterization and distribution of bHLH domains. The top: sequence logo of the FvbHLH domain by MEME. The bottom: distribution of amino acids in the bHLH consensus motif among strawberry. Asterisk symbol corresponds to each column above, which stands for the percentage of presence of amino acids at each site and the color of the asterisk symbol corresponds bHLH regions from the top insert. The analysis of the amino acids composition at each site marked by the asterisk indicates that the conservation of conserved amino acids is over 50%.

In order to know sequence features of strawberry bHLH domains and further to understand FvbHLH gene’s function, we performed multiple sequence alignment of amino acid sequences of the 113 strawberry bHLH (Figs 3; S1). It is revealed that there are four conserved regions for a bHLH domain, including one basic region, two helix regions and one loop region. We find that residues of His-2, Glu-6, Arg-7, Arg-9, Arg-10, Leu-20, Leu-23, Leu-36, Leu-46, etc., in the bHLH domain are conserved, implying that the amino acid residues may play an important role in strawberry’s evolution. In addition, we notice that the basic region of the bHLH domain can bind to DNA and it is critical to the gene biofunction^4^. It also has been known that both Glu-6 and Arg-9 in basic region of bHLH domain play important roles in the DNA binding^4,9,13^ and recognition of G-box and E-box (binding mode). As a result, we divided the FvbHLH binding into three modes: G-box (with the presence of His/Lys-2, Glu-6 and Arg-9), E-box (with the presence of Glu-6 and Arg-9) and non-E-box (without the simultaneous presence of Glu-6 and Arg-9) binding^42^. As is demonstrated in Fig. S1, FvbHLH proteins are divided into three types: 57 for the G-box-binding, 25 for the E-box-binding, 31 for the non-E-box-binding.

### Gene structure and conserved motif analysis of *FvbHLH* genes

Gene structure and conserved motif analysis of *Arabidopsis* and strawberry bHLH were performed to acquire more information about gene families^5^. By scanning all aspects of gene structure and conserved motif, genes within each subfamily are discovered to contain a similar number of intron and conserved motif, while the number of them is strikingly different on genes from different subfamily (Figs S2; S3), in consistent with the previous bootstrap analysis^43–45^. For instance, each gene from III(d + e) subfamily contains one exon except for *FvbHLH97* and *AtbHLH14* genes. In sharp contrast to this, 77.8% of *bHLH* genes from Ia subfamily contain three exons and two introns.

It has been pointed out that part of motifs, acting as activation domain, are important for the interaction with other modules of the transcription complex, and are the targets of signal transduction chains^5,10^. It might be inspiring to see how the motif structure is related to the gene classification. Thus, we searched 24 conserved motifs by MEME (Multiple Expectation Maximization for Motif Elicitation) program to obtain their distributions on bHLH sequences (Figs S2; S3). As is shown in Fig. S2, the bHLH proteins identified from the same subfamily share similar conserved motif. For example, motif 21 is exclusively located in all members from the XIII subfamily, whereas all bHLH sequences from IVc subfamily contain motif 1, motif 2, motif 10 and motif 15 at the C-terminal region. As bHLH is composed of motif 1 and motif 2, both of which are consistently identified in all strawberry and *Arabidopsis* bHLH proteins (Figs S2; S3). Hence, the classification of 26 subfamilies is thus further supported by the gene structure and motif analysis.

### Transcript patterns of *FabHLH* genes among different tissues in three cultivated strawberry varieties

**Figure 4:**
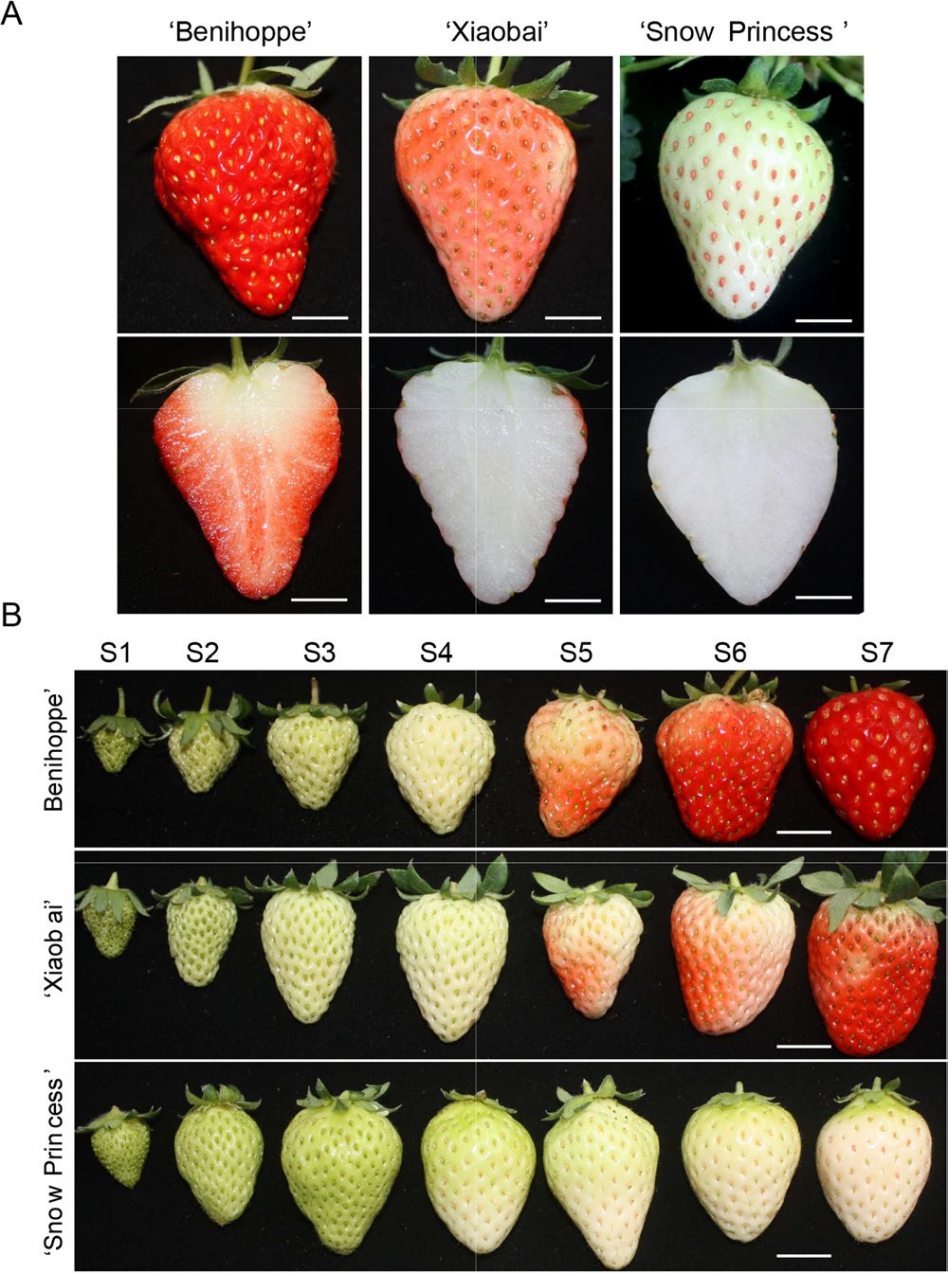
Materials of strawberry used in this study. (**A**) The fruit of ‘Benihoppe’, ‘Xiaobai’, and ‘Snow Princess’. (**B**) Seven fruit development and ripening stages of ‘Benihoppe’, ‘Xiaobai’, and ‘Snow Princess’. *Bar* = 1 cm.

**Figure 5:**
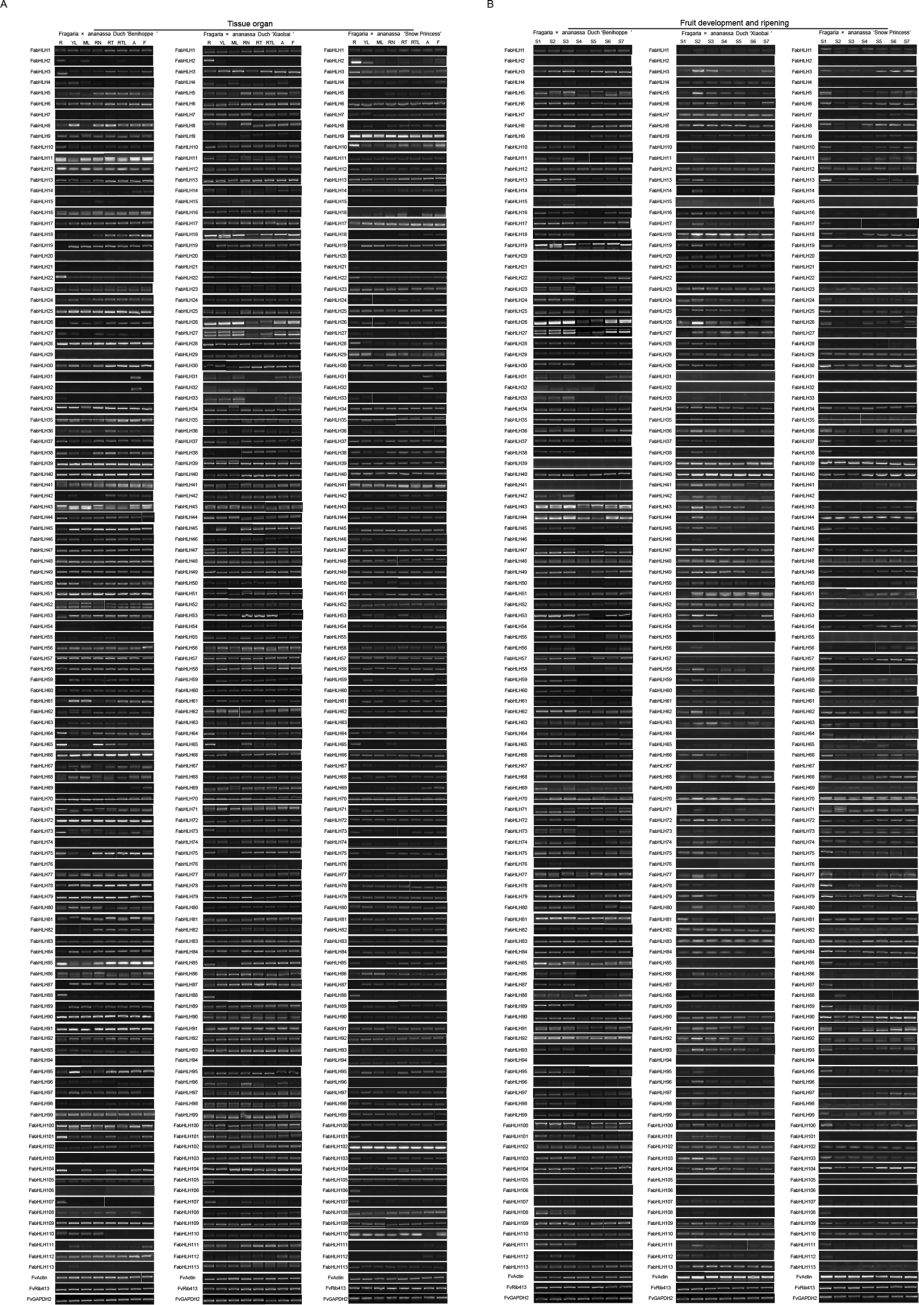
Transcript accumulation profiles of 113 *FabHLH* genes from different tissues and seven fruit development and ripening stages using semi-quantitative PCR for the three cultivated strawberry varieties. (**A**) Lanes: R, roots; YL, young leaves; ML, mature leaves; RN, runners; RT, runner tips; RTL, runner with tips and one leaf; A, anthotaxy; F, flowers. (**B**) Lanes: S1, small green fruit; S2, middle green fruit; S3, large green fruit; S4, white fruit; S5, initial red; S6, partial red; S7, full red. *FvActin*, *FvRib413* and *FvGAPDH2* were used as an internal control.

To reveal FabHLH genes (*F. ananassa* bHLH)’ role in regulating strawberry’s development, we focus on their temporal and spatial transcript patterns from eight different organs/tissues for three cultivated strawberry varieties (‘Benihoppe’, ‘Xiaobai’, and ‘Snow white’) under standard growth conditions (Figs 4A; 5A). We observe from the Fig. 5A that 78 *bHLH* genes are highly transcripted in certain tissues and their transcript patterns are similar to each other. For example, the *FabHLH31* and *FabHLH32* from IIIb subfamily are only transcript in anthotaxy for the two varieties (‘Benihoppe’ and ‘Snowwhite’); the *FabHLH6* and *FabHLH49* show similar transcript mode in all tissues for the three varieties (‘Benihoppe’, ‘Xiaobai’, and ‘Snowwhite’). However, some *bHLH* genes show observable different transcript behavior for the three varieties. For instance, *FabHLH5* carries on the same transcript pattern with certain degree of expression in tissues from ‘Benihoppe’ and ‘Xiaobai’, while it is barely expressed in ‘Snow Princess’, resulting into a considerable deviation from the transcript pattern for the other two. Specially, expression mode of *FabHLH18* differs for all the three varieties: highly transcripted in all tissues from ‘Xiaobai’, highly transcripted in some tissues from ‘Benihoppe’, lowly expressed in all tissues from ‘Snow Princess’.

### Transcript patterns of *FabHLH* genes during the fruit development and ripening for the white-flesh mutant strawberry

In order to identify bHLH genes involved in the color formation of strawberry fruit, three cultivated strawberry varieties were used in this study: Benihoppe, Xiaobai and Snow Princess. Colors of both the fruit flesh and skin of ‘Benihoppe’ are red. As the mutant of ‘Benihoppe’, ‘Xiaobai’ carries on the white or yellow color for its flesh with its fruit skin red or pink^31^. White is found for the color of ‘Snow Princess’ fruit flesh and skin (Fig. 4A). Additionally, strawberry fruit development and ripening are divided into seven stages: S1 small green fruit, S2 middle green fruit, S3 large green fruit, S4 white fruit, S5 initial red, S6 partial red, S7 full red (Fig. 4B). Because of the strong correlation between the gene expression pattern with its function, transcript patterns of 113 *FabHLH* genes for the color formation during the fruit development and ripening stages for the three varieties are tracked and summarized in Fig. 5B, in which the synthesis of anthocyanin is recorded from the turning stage to the red stage^28^. To examine the transcript of *FvbHLH* genes involved in the anthocyanin biosynthesis, both the RT-PCR (semi-quantitative reverse-transcription PCR) and qRT-PCR (quantitative RT-PCR) techniques are adopted to analyze genes’ expression level.

Figure 5B reveals that the number of up-regulated expression of *FabHLH* genes from ‘Benihoppe’ is 71 during the fruit ripening and this number from the ‘Snow Princess’ and ‘Xiaobai’ continuously falls down to 45 and 24, respectively. Depending on the consistency between the expression level of the up-regulated genes and anthocyanin content (Fig. 4B), 7 *FabHLH* genes are chosen out of the 113 genes to further investigate the possible expression patterns of bHLHs involved in the anthocyanin biosynthesis (Fig. 5): *FabHLH17, FabHLH25, FabHLH27, FabHLH29, FabHLH40, FabHLH80, FabHLH98*. In the following will be reported three relevant gene expression patterns: First, we will focus on the *FabHLH25*. Its expression is significantly up-regulated during all stages for ‘Benihoppe’ fruit, in accordance with its color of fruit skin and flesh, indicating that *FabHLH25* promotes the anthocyanin biosynthesis for ‘Benihoppe’; for ‘Xiaobai’ fruit, it is up-regulated at S2 stage and subsequently down-regulated at S5 stage, in discordance with the color of fruit skin while coinciding with the color of fruit flesh, suggesting that *FabHLH25* is not relevant to the anthocyanin biosynthesis for ‘Xiaobai’; however, the expression of *FabHLH25* is always down-regulated in the whole life for ‘Snow Princess’ fruit, agreeing well with the color of fruit skin and flesh, implying that *FabHLH25* is barely related to the anthocyanin biosynthesis for ‘Snow Princess’. As a consequence, expression level of *FabHLH25* shows significant difference between ‘Benihoppe’ and ‘Xiaobai’, and no observable difference between ‘Xiaobai’ and ‘Snow Princess’ is found from S4 to S7. This result implies that the *FabHLH25* might be involved in the anthocyanin biosynthesis for the fruit flesh. Second, we will turn to *FabHLH27* gene. Its expression is up-regulated during the overall stages for both the ‘Benihoppe’ and ‘Xiaobai’ fruits. This mode coincide with the color of fruit skin for ‘Benihoppe’ and ‘Xiaobai’ and the color of fruit flesh for ‘Benihoppe’, and is inconsistent with the color of fruit flesh for ‘Xiaobai’. The consistency here indicates that *FabHLH27* promotes the anthocyanin biosynthesis for both the ‘Benihoppe’ and ‘Xiaobai’. Nevertheless, *FabHLH27* gene’s expression is always down-regulated for ‘Snow Princess’ fruit, in perfect agreement with the color of fruit skin and flesh for ‘Snow Princess’, implying that *FabHLH27* is not in charge of the anthocyanin biosynthesis for ‘Snow Princess’. In brief, expression level of *FabHLH27* shows significant difference among ‘Benihoppe’, ‘Xiaobai’ and ‘Snow Princess’ from S4 to S7. This feature signifies that the *FabHLH27* could promote the anthocyanin biosynthesis for the fruit skin. Third, we will cast our eyes on the *FabHLH80* gene. Its expression is constantly down-regulated for ‘Benihoppe’ fruit, in good accordance with the color of fruit skin and flesh for ‘Benihoppe’, suggesting that *FabHLH80* is not involved in the anthocyanin biosynthesis for ‘Benihoppe’. *FabHLH80* gene’s expression is up-regulated at S2 stage and subsequently down-regulated at S5 stage for ‘Xiaobai’ fruit, going inversely with the color of fruit skin and flesh for ‘Xiaobai’, indicating that *FabHLH80* does not promote the anthocyanin biosynthesis for ‘Xiaobai’; nevertheless, *FabHLH80* becomes down-regulated at S2 stage and up-regulated at S4 stage for ‘Snow Princess’ fruit, in good accordance with the color of fruit skin and flesh for ‘Snow Princess’, implying that *FabHLH80* does not promote the anthocyanin biosynthesis for ‘Snow Princess’ either. As a short summarize, expression level of *FabHLH80* shows significant difference from S4 to S7 for three varieties. Such a mode leads us to the conclusion that the *FabHLH80* may inhibit the anthocyanin biosynthesis. Based on those observations and our more extensive data on expression patterns of the 7 previously selected bHLH genes, it is shown that they are indeed related to the anthocyanin biosynthesis.

**Figure 7:**
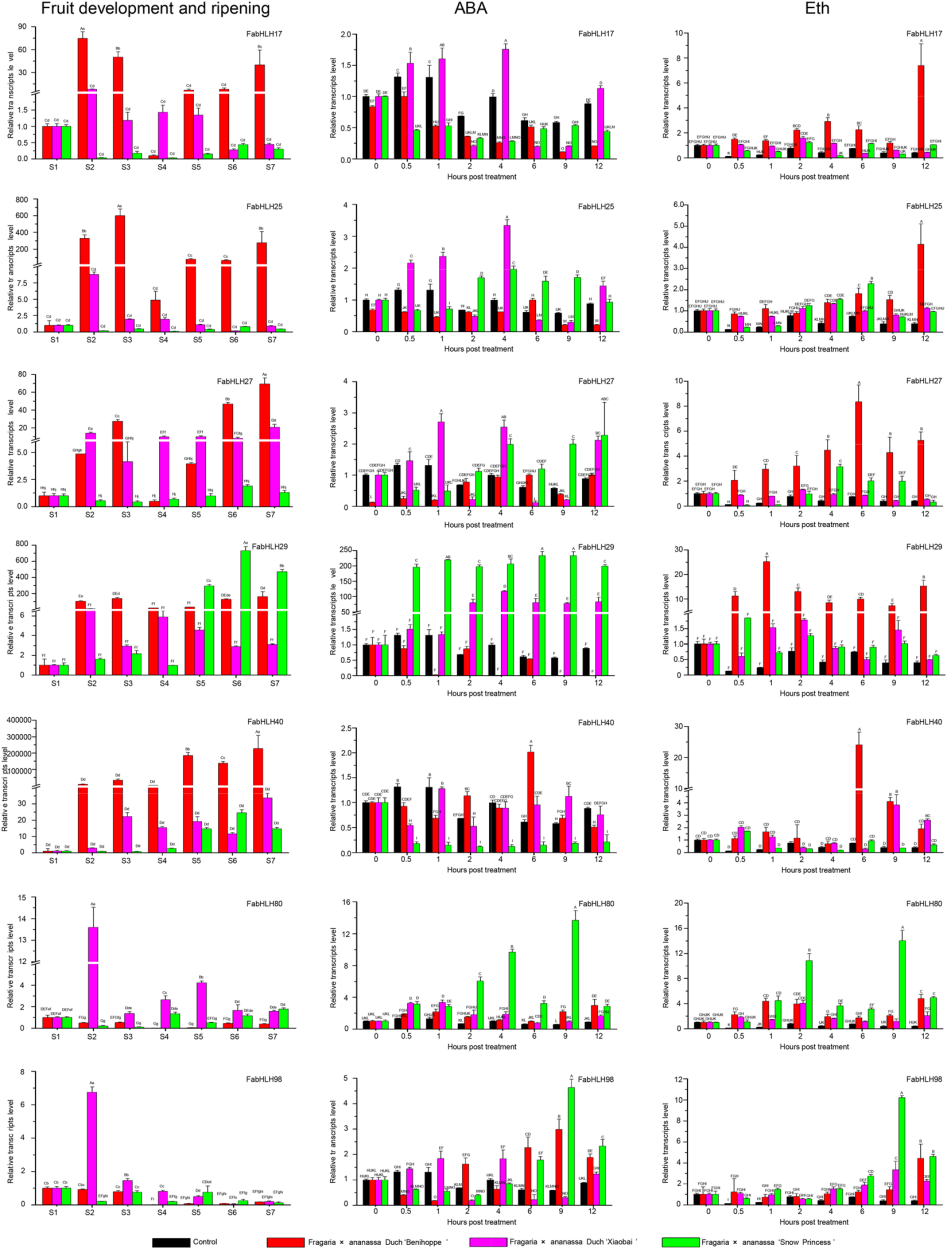
qRT-PCR transcription analysis of seven selected FabHLH genes during fruit development and ripening stages, and them under either ABA or Eth treatment for the three strawberry varieties. *FvRib413* is used as an internal control. The experiments were repeated three times and gave consistent results. The mean values and SDs were obtained from three biological and technical replicates. Different letters indicate the statistical difference among samples at P ≤ 0.01 and P ≤ 0.05 (fruit development and ripening), and P ≤ 0.01 (ABA and Eth treatments) according to Duncan’s multiple range test.

### Transcript patterns of the *FabHLHs* genes’ response to hormone treatment

**Figure 6:**
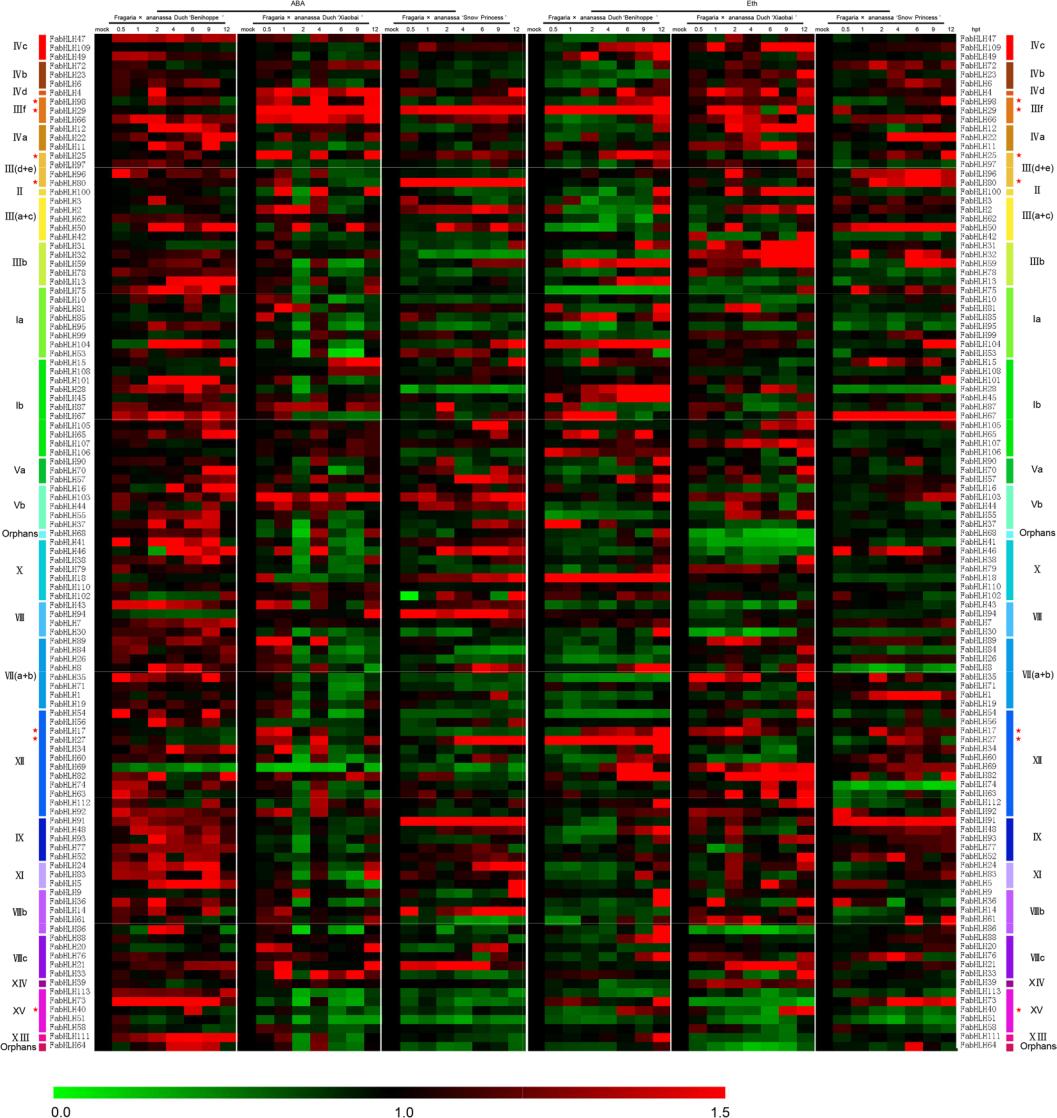
Transcript accumulation patterns of 113 bHLH genes for the three strawberry varieties under hormone stress (ABA and Eth). *FvActin*, *FvRib413* and *FvGAPDH2* were used as an internal control. The transcript accumulation profiles were generated by semi-quantitative PCR and were visualized as heat maps. The color scale represents the relative transcript level with increased (red) and decreased (green) transcript abundance. The FvbHLH genes marked by red asterisk indicate their candidacy in the anthocyanin biosynthesis.

Regarding to the fact that both ABA and Eth are critical plant hormone involved in the plant response to abiotic stress at the fruit ripening^9,46,47^, we further investigated responding transcript patterns of 113 *FabHLH* genes for the three varieties under the treatment of either ABA or Eth (Figs 6; 7; S4; S5). With the implement of ABA, numbers of responsive *FabHLH* genes from ‘Benihoppe’, ‘Xiaobai’ and ‘Snow Princess’ are 62, 47, and 43, respectively, in which 35 shared genes are founded for all the three. In parallel, numbers of responsive *FabHLH* genes from ‘Benihoppe’, ‘Xiaobai’ and ‘Snow Princess’ under the exposure to Eth are 67, 75 and 57, respectively, with a shared number of 34 for the three. For the two treatments, 25 genes are discovered to be simultaneously responsive for the three varieties. For example, the expression level of *FabHLH29* from IIIf subfamily strikingly increases at the initial stage (0.5 hpt (hour post treatment) to 2 hpt) and maintains a high value afterwards in response to ABA treatment for ‘Xiaobai’ and ‘Snow Princess’, while it decreases thoroughly under the ABA treatment for ‘Benihoppe’. When subjected to the Eth, *FabHLH29* expresses highly for ‘Benihoppe’ and keeps relatively low yet higher than the control for both ‘Xiaobai’ and ‘Snow Princess’. In addition, expression level of *FabHLH98* from IIIf subfamily is invariably high for the three varieties under both treatments compared with the control: the increase of it is significantly induced at early stages (0.5 hpt to 2 hpt), and it reaches the peak at later stages (4 hpt to 9 hpt) in response to the ABA treatment for ‘Benihoppe’ and ‘Xiaobai’. However, it is induced and starts to reach its maximum from 6 hpt to 9 hpt in response to ABA treatment for ‘Snow Princess’; under the treatment of Eth, *FabHLH98* ‘s expression is induced and begins to reach the peak at later stages (4 hpt to 12 hpt) for the three varieties. Besides, *bHLH* genes from III(d + e) subfamily are realized to be responsive to both treatments for the three varieties as well. This finding demonstrates that subfamilies of III(d + e) and IIIf might be involved in the fruit ripening and plant response to abiotic stress.

### Network interaction analysis of FabHLHs response to anthocyanin biosynthesis and hormone stress

**Figure 8:**
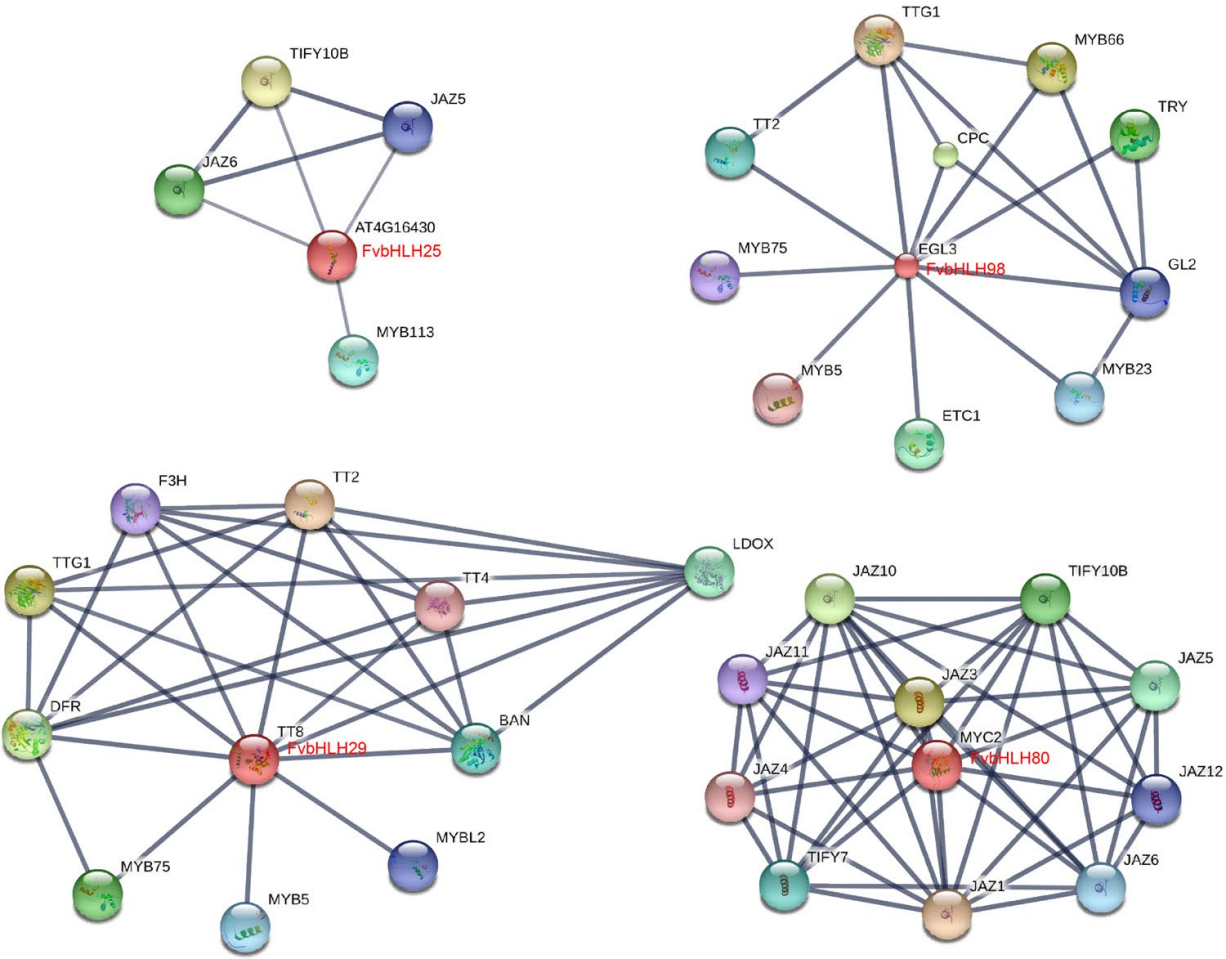
Interaction network analysis of bHLH proteins identified for strawberry and related genes for *Arabidopsis*. Line thickness is related to the combined score (FvbHLH25 score >0.7, the others score >0.9). The homologous genes of strawberry are in red.

The above results argue that 7 *FabHLH* genes are highly possible to be involved in the anthocyanin biosynthesis and hormone response pathway for strawberry as a result of the interaction between bHLH and other proteins. Network interaction analysis has been recently demonstrated to be a powerful method to study the gene function. Online software of STRING 10 is used to reconstruct the interaction network of the 7 FvbHLH based on the orthologous gene of *Arabidopsis*. Only 4 bHLHs (FvbHLH25, FvbHLH29, FvbHLH80, and FvbHLH98) are proved to be able to predict the interacting proteins (Fig. 8; Table S2). According to the database of STRING 10, they are involved in the control of flavonoid pigmentation, epidermal cell fate specification and regulation of ABA-inducible genes under drought stress conditions. As is shown in Fig. 8; Table S2, FvbHLH25 (homologous to AT4G1640 for *Arabidopsis*) can be associated with MYB113, which could combine with several bHLH proteins in the anthocyanin biosynthesis^48^. Besides, FvbHLH25 also interacts with JAZ5 (JASMONATE ZIM-Domain 5) and JAZ6, which are the repressor of jasmonate response. FvbHLH29 (homologous to TT8 for *Arabidopsis*) can interact with MYB75, which promotes the synthesis of anthocyanin biosynthesis by activating the expression of DFR (dihydroflavonol-4-reductase) such that it is eventually involved in the control of flavonoid pigmentation. Moreover, FvbHLH80 (homologous to MYC2 for *Arabidopsis*) could react with MYB2 in the regulation of ABA-induced genes under drought stress conditions, as well as with MYC3 and MYC4 in the control of subsets of JA-dependent responses. In addition, FvbHLH98 (homologous to EGL3 for *Arabidopsis*) participates in the anthocyanin accumulation in *Arabidopsis*^1,48,49^ and tomato^21^. These results show that 4 FvbHLHs are involved in the fruit ripening and hormone response pathway^25,34,38,47,50^.

## Discussion

With the functionality being the transcription, bHLH family are involved in the regulatory process of fruit ripening, hormone signaling and abiotic stress^12^. In the past few decades, features and functions of the bHLH gene family have been identified and investigated for several plant species^3,8,12^. Though as one of the most important horticultural crops grown worldwide providing ingredient for processed foods like jams and juices, strawberry has been barely studied for its bHLH family, who participates in the anthocyanin biosynthesis in the fruit ripening. Very few bHLHs have been investigated for the strawberry, such as *FabHLH3*^38^, *FaSPT* (spatula)^40^ and *FvbHLH33*^39^. In the present study, we first identified a total of 113 bHLH genes based on the *F. vesca* genome (Table 1 and Fig. 1), and further implemented their bioinformation analysis (Figs 2; 3; S2) followed by the expression pattern classification during the fruit ripening under hormone treatments for three varieties (Figs 5; 6; 7).

With the rapid development of bioinformation analysis, the information stored in various genomes can be decoded to elucidate mechanisms that regulate fruit ripening and response to abiotic stress^4^. We firstly identified 113 unique bHLH proteins using the conserved motif of bHLH by filtering candidate genes according to the criteria described by Sun *et al*.^3^. Next, based on the phylogenetic analysis of FvbHLH, the selected FvbHLHs were classified into 26 subfamilies (Fig. 3) with the methodology similar to the classification of *Arabidopsis* (26 subfamilies), tomato (26 subfamilies) and Chinese cabbage (26 subfamilies)^2–4,13^. Moreover, the analysis of motif and gene structure is performed to gain evidence to support phylogenetic relationship for gene families.

Most bHLH proteins identified so far are mostly functionally characterized for *Arabidopsis* and tomato, with the revealing of their effects on the regulation of plant development, fruit ripening, anthocyanin biosynthesis and hormone signaling responses^6,16^. Those results prove that transcript pattern of a gene is closely related to its function, based on which we designed to examine the expression patterns of 113 *FvbHLH* genes from tissues, at fruit ripening stage, as well as those under the treatment of hormone (Figs 5; 6; 7). We discover that the expression patterns for the 78 out of the 113 genes from various tissues for the three varieties are similar to each other. To comprehensively understand the role of bHLH genes on the anthocyanin biosynthesis, RT-PCR and qRT-PCR analyses for the three varieties with different fruit flesh and skin colors were performed (Figs 4; 5B; 6; 7). 7 *FabHLHs* are found to be highly responsive for the anthocyanin biosynthesis depending on their different expression levels: *FabHLH17*, *FabHLH25*, *FabHLH27*, *FabHLH29*, *FabHLH40*, *FabHLH80*, *FabHLH98*. For example, the expression level of *FabHLH27* is high for both ‘Benihoppe’ and ‘Xiaobai’ (red or pink skin) at the later stages (S5 → S7), while it stays low for ‘Snow Princess’ (white skin) at the similar stage S5. This implies that this gene is involved in the anthocyanin biosynthesis of fruit skin.

It has been reported that IIIf subfamily matters for the fruit color formation. Hereby, we focus on the 2 out of the 7 candidate *FabHLHs* that fall into the IIIf subfamily: *FabHLH29 and FabHLH98*. We found that *FabHLH29* is relevant to the anthocyanin biosynthesis according to its expression pattern during the fruit ripening for the three varieties. Besides, gene sequence of *FabHLH29* is highly similar to that of AtTT8 (AtbHLH42), which has been reported to be involved in anthocyanin biosynthesis^1,6,15^. Moreover, the *FabHLH29* also is responsive to both the ABA and Eth treatments, thought with certain difference (down-regulated for ‘Benihoppe’ under ABA treatment, up-regulated for rest cases), for the three varieties. More evidence for the involvement *FabHLH29* in the anthocyanin biosynthesis comes from the interaction network. Proteins (F3H (Flavanone 3-hydyroxylase), DFR, TTG1 and MYB), located in the pathway of anthocyanin biosynthesis, are predicated to interact with *FabHLH29* (AtTT8) (Fig. 8). Researchers have realized that the TT8 from subfamily IIIf is active in regulating the synthesis of anthocyanin and proanthocyanidin for *Arabidopsis*^1,6,50,51^ by forming a stabilized MBW complex with TT2 and TTG1, and it is involved in the anthocyanin biosynthesis for rice as well^22^. We also find that the expression pattern of *FabHLH98* (homologous to EGL3) shows no significant difference during the fruit ripening for ‘Benihoppe’, ‘Xiaobai’, and ‘Snow Princess’, which denies the participation of *FabHLH98* in the anthocyanin biosynthesis. However, *FabHLH98* is responsive to the abiotic stress with the implement of ABA and Eth, which seems to suggest its involvement in the fruit ripening. What’s more, analysis of interaction network of *FabHLH98* demonstrates that it also plays a role in the activation of anthocyanin biosynthesis, possibly with MYB75/PAP1, inconsistent with previous results from the analysis of expression pattern during the fruit ripening in this study, yet in good agreement with the precursor reports^1,6,27^. In brief, expression pattern analysis under hormone treatments fits well with results from the interaction network investigation for the three varieties. However, both are inconsistent with expression pattern results during the fruit ripening. Consequently, *FabHLH98* is selected as the candidate gene for the study of anthocyanin biosynthesis and a further study on its precise role is still in demand.

Previous papers inform that genes from bHLH subfamily III(d + e) take part in JA signal pathway, resulting into the regulation of plant defense during developmental process for *Arabidopsis*^23,25,26^ and the promotion of anthocyanin biosynthesis^24,27^ for apple. Moreover, the function of bHLH subfamily IIId, including bHLH3, can negatively regulate JA-mediated plant defence and development^13^, while the function of bHLH subfamily IIIe can activate JA-induced leaf senescence^25^. In addition, as a repressor in the JA signaling pathway, MdJAZ can be phosphorylated by MdSnRK1.1 (Snf1-Related protein Kinases) to facilitate its 26S proteasome-mediated degradation, releasing MdbHLH3 which will bind to promoters of the anthocyanin biosynthesis genes *MdDFR* and *MdUFGT*, thus finally promotes the biosynthesis of anthocyanin and proanthocyanidin^24,27^. In our experiments, we find that *FabHLH25* from III(d + e) subfamily might be correlated with the anthocyanin biosynthesis of fruit flesh (Figs 5B, 7) from the analysis of the expression pattern for the three varieties during their ripening. Moreover, the FabHLH25 (homologous to AT4G16430, FabHLH3 and MdbHLH3) protein strongly interact with MYB113, JAZ5 and JAZ6 proteins (Fig. 8) according to results from interaction network analysis, in consistent with the known knowledge that FabHLH25 is able to interact with MYB and form the MBW complex to regulate the expression of genes involved in the proanthocyanidin biosynthesis^38^. What’s more, it has been mentioned that MdMYC2 positively regulates anthocyanin biosynthesis by modulating the expression of positive regulators in JA signaling (MdMYB1, MdbHLH3, MdbHLH33) for the apple^52^. From our observation, the transcript pattern and interaction network analysis evidence that the *FabHLH80* (homologous to MYC2) from III(d + e) subfamily might also be present in the anthocyanin biosynthesis. Therefore, our research hereby paves the way for further studies and understandings of bHLH genes function in the fruit ripening and anthocyanin biosynthesis for strawberry.

In conclusion, the first comprehensive and systematic analysis of strawberry bHLH transcription factors is performed. First, 113 bHLH transcription factors from the entire strawberry genomes are identified as candidate genes responsible for the anthocyanin biosynthesis and further renamed based on their chromosome distribution. Next, the selected genes are divided to 26 subfamilies according to phylogenetic analyses, gene structures and protein motifs. Third, expression patterns of 113 *FabHLHs* obtained during fruit development and ripening, as well as those under either the ABA or Eth treatment, suggest that seven *FabHLHs* (*FabHLH17*, *FabHLH25*, *FabHLH27*, *FabHLH29*, *FabHLH40*, *FabHLH80*, *FabHLH98*) are involved in the anthocyanin biosynthesis of strawberry fruit. Finally, results of interaction network analyses of the four *FabHLH* genes (*FabHLH25*, *FabHLH29*, *FabHLH80*, *FabHLH98*) reveal that bHLHs proteins might participate in the anthocyanin biosynthesis during the fruit ripening and in the hormone response pathway. This study will provide an insight into a further understanding of functions of bHLH members in the color formation for fruits.

## Materials and Methods

### Identification of bHLH transcription factors for strawberry

To identify bHLH transcription factors in the strawberry genome (*F. vesca*), we performed a search from the NCBI database (*F. vesca* (taxid:57918)) (https://www.ncbi.nlm.nih.gov/genome/3314). The published *Arabidopsis* and strawberry bHLH protein sequences were downloaded from the Plant Transcription Factor Database (http://planttfdb.cbi.pku.edu.cn/) and used as queries in BLAST-P searches with default parameters in NCBI database. To further validate all bHLH transcription factors, full-length amino acid sequences of the 166 putative candidates were verified using the CDD (https://www.ncbi.nlm.nih.gov/Structure/cdd/wrpsb.cgi), the hidden Markov model of SMART (http://smart.embl-heidelberg.de/smart/set_mode.cgi?NORMAL=1)^53,54^ and InterProScan program (http://www.ebi.ac.uk/inter-pro/search/sequence-search) to confirm their completeness and the presence of bHLH domain. Details about the bHLH sequences, such as length of amino acid sequences, theoretical molecular weights (Mw) and isoelectric point (pI), were obtained from ExPASy Proteomics server (http://web.expasy.org/compute_pi/).

### Bioinformatic analysis of *bHLH* transcription factors for strawberry

Chromosomal localization data was retrieved from NCBI Map Viewer (https://www.arabidopsis.org/mapview/). Genes were mapped to the chromosomes using MapDraw. These genes were renamed from *FvbHLH1* to *FvbHLH113* according to their position, from the top to bottom, on the *F. vesca* chromosome^8,41^. Multiple domain alignments of strawberry bHLH proteins and domains were performed using ClustalX 2.0.12 with default settings for obtained sequences of the FvbHLH domains, and alignment results were shown and drew by OriginPro 8^9^. To compare the evolutionary relationship between *Arabidopsis* (AtbHLH) and strawberry (FvbHLH), we obtained the phylogenetic tree for bHLH proteins using MEGA5.1 with the neighbor-joining method and the following parameters: complete deletion, p-distance model and 1000 replicates of bootstrap method^4,9^. 26 subfamilies were identified according to the clade support values, topology of the trees, branch lengths, visual inspection of the bHLH amino acid sequences and classification of strawberry^2,4,10^. The online Gene Structure Display Server (GSDS 2.0, http://gsds.cbi.pku.edu.cn/) was used to investigate the exon-intron structure of the FvbHLH transcription factors based on each coding sequence (CDS) and corresponding genomic sequence. Conserved motifs in FvbHLH transcription factors were identified from the online MEME (http://meme-suite.org/tools/meme). The FvbHLH25, FvbHLH29, FvbHLH80 and FvbHLH98 protein sequences were employed as queries for the BLAST-P search in *Arabidopsis* Information Resource (TAIR, https://www.arabidopsis.org/) to obtain protein sequences of AT4G16430, AtTT8, AtMYC2 and AtEGL3, respectively. Specific interaction network with experimental evidences of AT4G16430, AtTT8, AtMYC2 and AtEGL3 was constructed using online STRING 10 (http://string-db.org/) with option value >0.700 or 0.900.

### Plant materials, growth conditions and treatments

Three octoploid cultivated strawberry varieties (*F. ananassa* Duch. ‘Benihoppe’; *F. ananassa* Duch. ‘Xiaobai’, the white-flesh mutant of ‘Benihoppe’; *F. ananassa* ‘Snow Princess’ with white fruit skin and flesh.) were used in this study (Fig. 4A). Plantlets of the three varieties were grown in the strawberry germplasm resource greenhouse of Zhengzhou Fruit Research Institute, Chinese Academy of Agricultural Sciences, Zhengzhou, Henan, China (Fig. 4A). Strawberry plantlets were transplanted into a plastic pot (diameter: 17 cm, height: 15 cm) containing soil mix (perlite: peat, 1: 4, v/v) and grown in greenhouse with temperatures ranging from 8 °C to 28 °C, relative humidity ranging from 55% to 70%, and without supplemental lighting.

To analyze transcript patterns of bHLH transcription factors, strawberry organs/tissues (roots, young leaves, mature leaves, runners, runner tips, runner with tips and one leaf, anthotaxy, flowers, small green fruit, middle green fruit, large green fruit, white fruit, initial red fruit, partial red fruit, full red fruit) were obtained from different developmental stages. Various vegetative and reproductive tissues were collected and stored at −80 °C for tissue-specific experiments. To analyze the expression level of bHLH transcription factors to different hormones, strawberry plantlets at the stage of the sixth leaf fully expanded were sprayed with ABA at 0.1 mM, Eth at 0.5 g/L, and water, respectively. Leaf samples were collected for RNA extraction at 0, 0.5, 1, 2, 4, 6, 9 and 12 hpt. Leaves with water treatment at 0 hpt were used as control. Each time for each treatment, one leaf from each of the three separate plants, thus three leaves in total, was picked up to conduct one analysis, and all treatments were performed thrice independently.

### RNA preparation, semi-quantitative reverse-transcription PCR and quantitative real-time PCR Analysis

Each RNA was extracted from tissue samples using the E.Z.N.A Plant RNA Kit (Omega, China) according to the manufacturers’ instruction. RNA concentration and quality were measured by the NanoDrop 1000 (Thermo, USA). The first-strand cDNA was synthesized using the PrimerScript^TM^ RT reagent Kit with gDNA Eraser (TaKaRa, China) according to the manufacturers’ instruction. The concentration of cDNA was adjusted based on the strawberry housekeeping genes *FvActin*, *FvRib413* and *FvGAPDH2*^44,46^. The primers used in this study were designed by the Vector NTI software (Table S1) without any interference with the conserved region, and were amplified the product to a length of of 150 bp to 300 bp. RT-PCR reactions were performed using 2 × Taq Mix (Beijing, China) with the following parameters: annealing temperature between 53 °C and 57 °C with 32–34 cycles. The PCR products were placed on the 1% (w/v) agarose gel with GelStain (10000×) (Tiangen, China) staining and further imaged under the AlphaView SA software. Each reaction was repeated three times. The expression data from the RT-PCR were acquired, analyzed, and visualized using the software AlphaView SA and Mev 4.8.1^43–45^. qRT-PCR was performed according to Wei *et al*.^46^. The primers were listed in the Supplemental Table S1.

### Statistical analysis

Statistical analysis was performed by the Duncan’s multiple range test module in the SPSS Statistics 17.0 software. Each experiment was independently repeated at least three times. Mean values ± standard deviation of the mean (SD) were presented (Fig. 7), and least significant differences were calculated at the 5% or 1% level of probability.

## Electronic supplementary material

Supplemental Figures

Supplemental Tables
