# Supplementary material for: Identification of basic/helix-loop-helix transcription factors reveals candidate genes involved in anthocyanin biosynthesis from the strawberry white-flesh mutant
Source: Sci Rep. 2018 Feb 9;8:2721. doi: 10.1038/s41598-018-21136-z (PMC5807450; doi:10.1038/s41598-018-21136-z)

Identification of basic/helix-loop-helix transcription factors reveals candidate genes involved in anthocyanin biosynthesis from the strawberry white-flesh mutant

Fengli Zhao[1,2], Gang Li[1,2], Panpan Hu[1,2], Xia Zhao[1,2], Liangjie Li[1,2], Wei Wei[3], Jiayue Feng[3], Houcheng Zhou[1,2*]

Supplemental materials

Supplementary Figure 1. Multiple sequence alignment of the bHLH domain among *FvbHLH* genes.

Supplementary Figure 2. Structural analysis of strawberry bHLH transcription factors. The gene structure is shown on the left, with exons and untranslated regions (UTRs) represented by blue-corner rectangles and red rectangles, respectively. The protein motif analysis of strawberry bHLH genes are shown on the right. 1-24 motif was denoted by rectangle with different colors on the lower right corner.

Supplementary Figure 3. Sequence logo of the FvbHLH domain by MEME.

Supplementary Figure 4. Analysis of semi-quantitative RT-PCR for expression profiles of 113 FvbHLH genes in response to ABA treatment. *FvActin*, *FvRib413* and *FvGAPDH2* were used as an internal control.

Supplementary Figure 5. Analysis of semi-quantitative RT-PCR for expression profiles of 113 FvbHLH genes in response to Eth treatment. *FvActin*, *FvRib413* and *FvGAPDH2* were used as an internal control.

Supplemental Figure 1

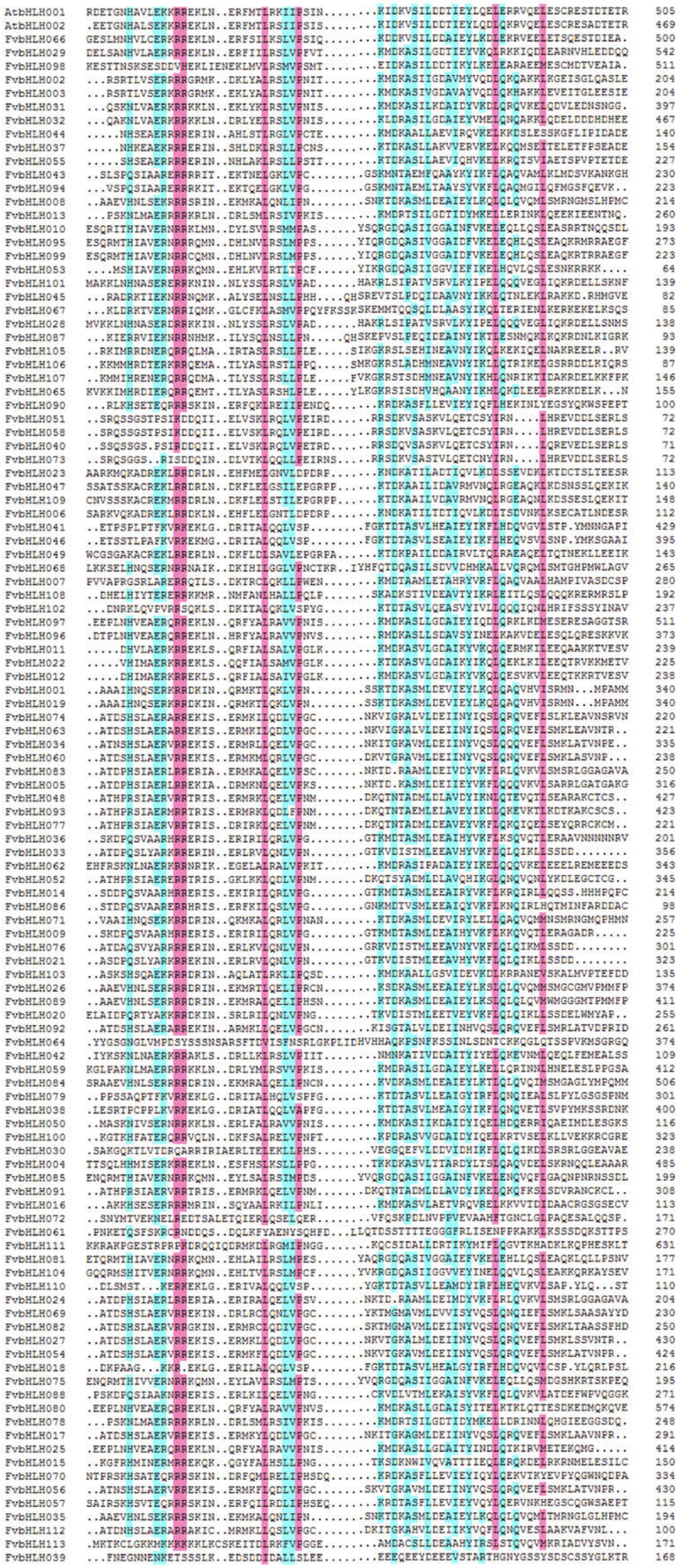

Supplemental Figure 2

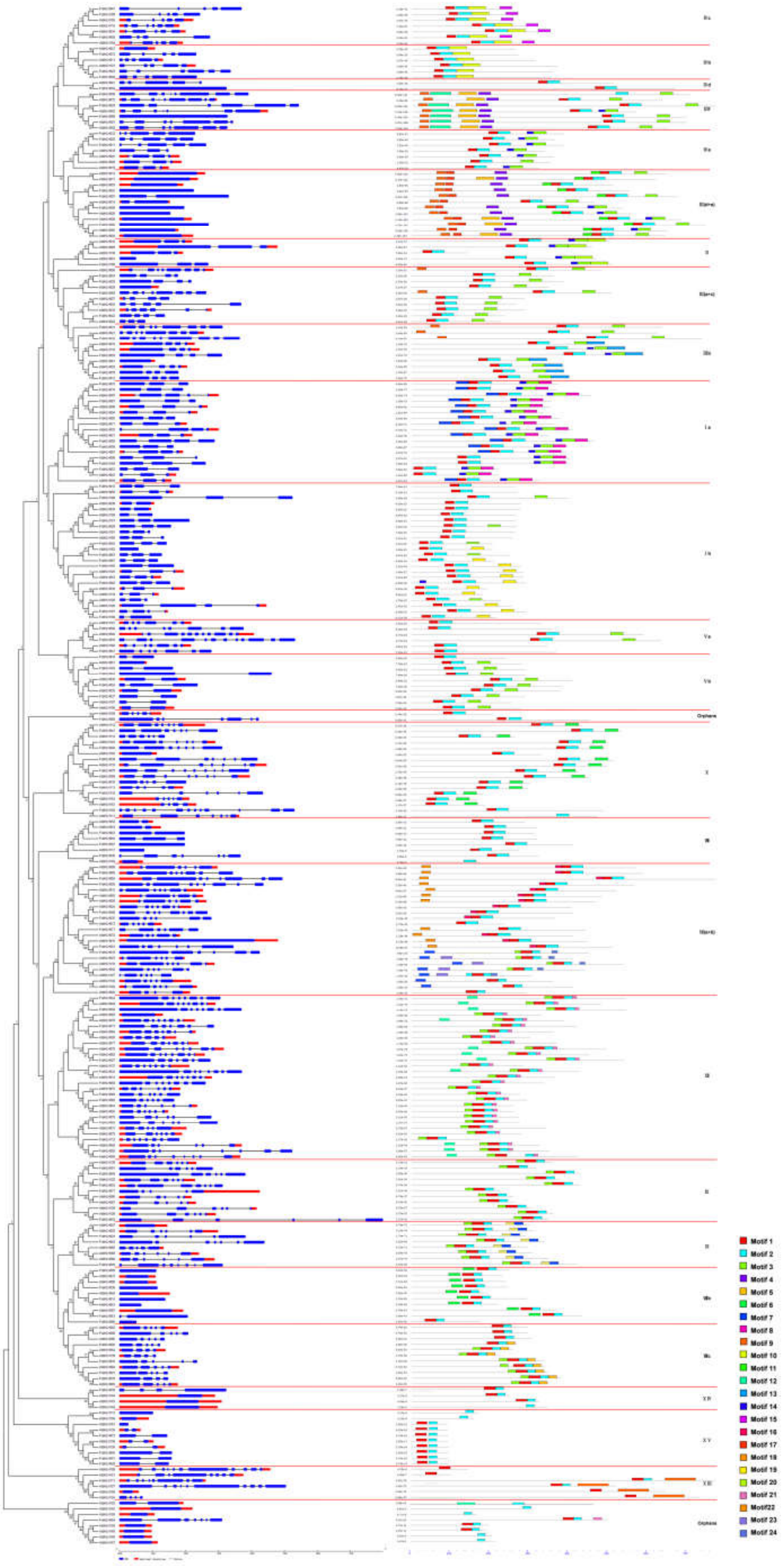

Supplemental Figure 3

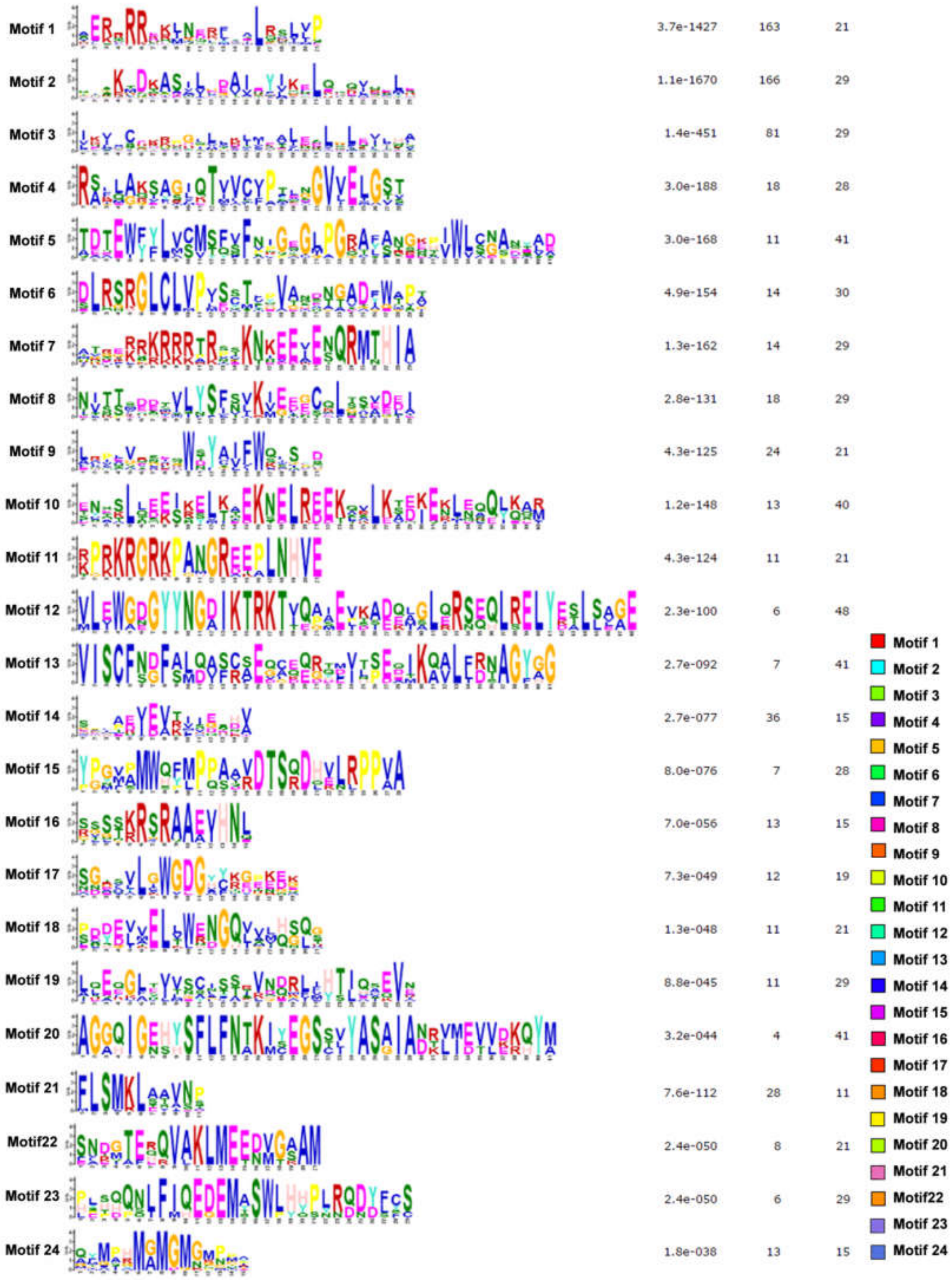

Supplemental Figure 4

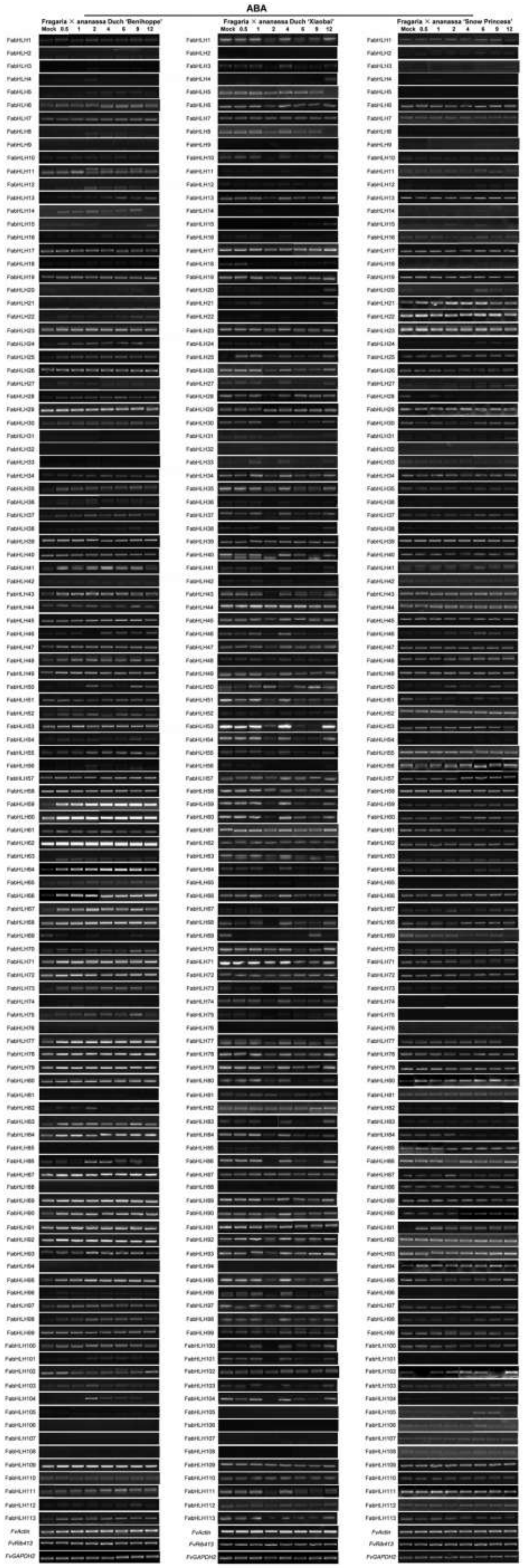

Supplemental Figure 5

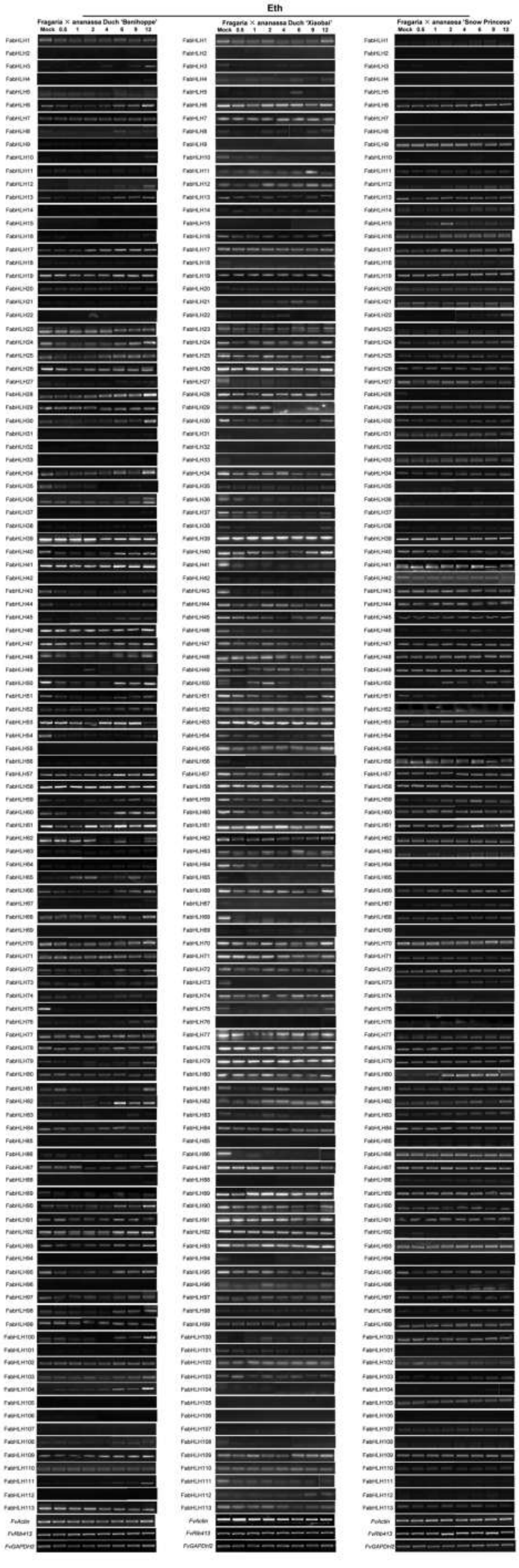

Supplement: Supplementary file 1 — Supplemental Figures [file 41598_2018_21136_MOESM1_ESM.pdf]
